# Surgery

**Published:** 1890-12

**Authors:** John Parmenter


### Progress in Medical Science.

### SURGERY.

CONDUCTED BY

JOHN PARMENTER, M. D.

### Dry Treatment of Open Wounds and Ulcers.

Byford (*Annals of Surgery*, October, 1890,) contributes an original memoir upon the Dry Treatment for Open Wounds and Ulcers. He made experiments in order to find "a method of cure for open wounds that would operate somewhat in the same manner as the closure and healing of incised wounds by primary union, viz., without sepsis and without suppuration.". He discarded germicides, and strove to imitate nature by drying the wound, and keeping it so, or when this was impossible to drain off the fluid so constantly that the same fluid would not remain in contact with the diseased surfaces, and thus allow the development of germs in it. The method was applied to suppurating wounds, as well as fresh, and consists in having abundant capillary drainage from *every* part of the wound, the drainage material to be changed before saturation is reached. Coincident with each dressing, the neighboring surfaces are cleaned, the wound dried, and new material for drainage reapplied. The author then gives the detail of six cases, comprising an abdominal oöphorectomy for abscesses of Fallopian tube and ovary, removal of suppurating kidney by laparatomy, mole removed from temple, bed sore, abscess of abdominal wall following laparatomy, and suppuration following Alexander's operation, in all of which the method worked to his complete satisfaction. He uses it constantly in fresh wounds, where it is applicable, in laparatomies after the second or third days, gauze replacing the drainage-tube, and for venereal sores between the times of cauterization. The dressings require to be changed oftener in suppurating wounds than in fresh, the intervals varying from every four to every six, eight or twelve hours according to the circumstances of the case. Gauze is preferable to absorbent cotton in large wounds. The latter, however, is applicable to most cases. The author sums up as follows :

1. Secure a large external opening.
2. Change the dressing often enough to prevent an accumulation of moist discharge.
3. Dry off the surfaces at each dressing as perfectly as possible.

4. Place absorbent material firmly against every part of the raw surfaces, but leave the packing loose in the middle so that the cavity may more readily contract.

5. Place an abundance of absorbent material over the wound, so as to be in direct contact with the packing, no powder or drug intervening.

6. Use clean absorbent cotton for small wounds, gauze for large ones.

7. Cleanse the neighboring skin at each dressing with dry absorbent material, or wash it with alcohol, but allow no water, or watery solution, to come in contact with the wound or its surroundings.

---

Laparatomy for Peritoneal Tuberculosis.—Lauenstein (*Centralblatt für Chirurg.*, 42, 1890,) offers some suggestions upon the rationale of laparatomy for the cure of peritoneal tuberculosis. He thinks the withdrawal of the coëxisting ascetic fluid stops the further progress of the malady, for the reason that the peritoneum is made dry, which condition is incompatible with the development and existence of the tubercle bacilli, and that, therefore, the cure of the tuberculosis, and the non-appearance of the ascites after laparatomy, stand in direct relationship. Furthermore, Koch's experiments show that sunlight quickly kills all bacteria growing upon a culture medium, and the tubercle bacilli in from a few minutes to one hour. The author then argues further that, inasmuch as a thorough examination of the abdominal cavity requires a good light, and that many cases are operated upon at a time of day when the light is the greatest, and that the operation room is usually very light, the direct action of sunlight may exercise a curative influence along with drainage. This supposition gets some support from the fact that in no other form of tuberculosisis is incision and drainage followed by cure, and in no other form is direct sunlight used as in laparatomy.

The author concludes with the narration of a case of peritoneal tuberculosis, in which he made laparatomy, drained off a large amount of ascetic fluid, and wiped out the cavity without using antiseptics. He then allowed sunlight to fall in every part of the cavity for ten minutes. The fluid did not reappear, and the patient remains well now, two months after operation.

---

Local Anesthesia.—Dr. A. Dobisch, of Zwittan, recommends spraying the parts for one minute to induce local anesthesia, with the following: chloroform, 10.00; ether, 15.00; menthol, 1.00. The anesthesia is complete, and lasts from two to six minutes.—*International Journal of Surgery*.

